# Management of Bilateral Ectopic Pregnancies after Ovulation Induction Using Unilateral Salpingectomy and Methotrexate for the Remaining Ectopic with Subsequent Intrauterine Pregnancy

**DOI:** 10.1155/2018/7539713

**Published:** 2018-07-05

**Authors:** Quinton Katler, Lindsey Pflugner, Anjali Martinez

**Affiliations:** Department of Obstetrics and Gynecology, The George Washington University Hospital, Washington, DC, USA

## Abstract

Bilateral ectopic pregnancy is a rare phenomenon which is found with increased frequency when using assisted reproductive technology (ART). This diagnosis is most often made incidentally and intraoperatively, as ultrasound and serial *β*-hCG trends have shown poor efficacy for accurate diagnosis. Management of bilateral ectopic pregnancies is most commonly reported using bilateral surgical removal of the ectopic pregnancy (salpingostomy and/or salpingectomy). We present a case of an ART patient with incidentally found bilateral tubal ectopic pregnancies, where multiple management strategies including medical and surgical techniques were used concurrently which resulted in a subsequent spontaneous intrauterine pregnancy. While the standard of care is difficult to establish, we recommend individualizing management decisions based on the patient's reproductive goals and overall risk profile.

## 1. Introduction

Unilateral ectopic pregnancy is a well-known and common diagnosis in the general population [1]. Conversely, bilateral ectopic pregnancy is a much rarer phenomenon, occurring in approximately 1 per 200,000 live births [2]. While bilateral ectopic pregnancies have been documented in the literature since the 1900s, the invention of assisted reproductive technologies (ART) has led to an increase in reported cases of bilateral ectopic pregnancies [3, 4].

Given the relative frequency of bilateral ectopic pregnancies in the ART population, the importance of choosing an appropriate management strategy is further underscored given the known morbidity and potential mortality of ruptured ectopics within the context of the patient's reproductive goals. There are established criteria for management of unilateral ectopic pregnancies, which include pharmacologic, surgical, and expectant management under specific circumstances. However, there are no well-defined studies or data to suggest standard of care in the case of bilateral tubal ectopic pregnancies, particularly in the setting of one ruptured and one nonruptured ectopic pregnancy. Upon review of the existing literature, there are no reports of medical and surgical management being used simultaneously for the management of bilateral ectopic pregnancies, nor are there reports of a subsequent spontaneous intrauterine pregnancy following this treatment approach. Here we present a case of an ART patient with incidentally found bilateral tubal ectopic pregnancies, where two different management strategies were used concurrently with a successful outcome.

## 2. Case Report

A 32-year-old G2P0020 healthy Caucasian female initially presented to our institution for outpatient evaluation and management of secondary infertility. Her obstetric history was notable for two first-trimester miscarriages that were both managed expectantly. The couple's infertility evaluation revealed normal ovarian reserve testing and semen-analysis parameters with an unremarkable hysterosalpingogram (HSG) study, and they were diagnosed with unexplained infertility. The patient underwent ovulation induction with clomiphene citrate and HCG trigger with timed intrauterine insemination (IUI) using her partner's sperm. In the weeks following IUI, the *β*-hCG level rose appropriately from 641 to 971 in 48 hours. One week later, the *β*-hCG level rose to 3,448 and TVUS revealed a small, irregularly shaped gestational sac in the uterus without a clear yolk sac or evidence of a fetal pole. The right adnexa appeared to have two corpus luteal cysts. Of note, no free fluid was identified in the cul-de-sac and the patient was asymptomatic at that clinic visit. The plan was for a repeat *β*-hCG level and TVUS in 48 hours.

**Figure 1:**
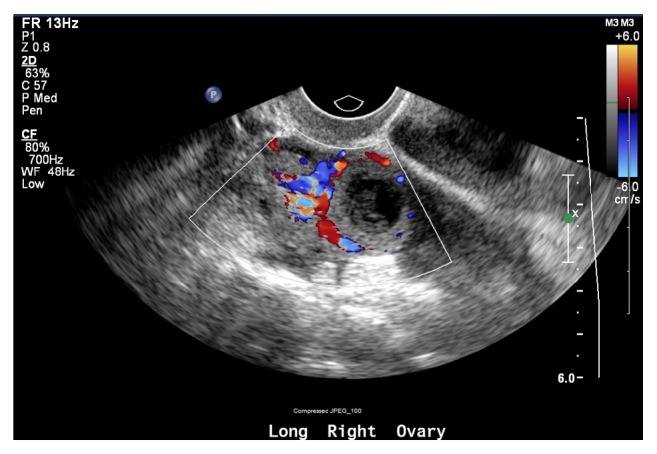
TVUS image of right adnexa with corpus luteal cyst versus ectopic gestation.

**Figure 2:**
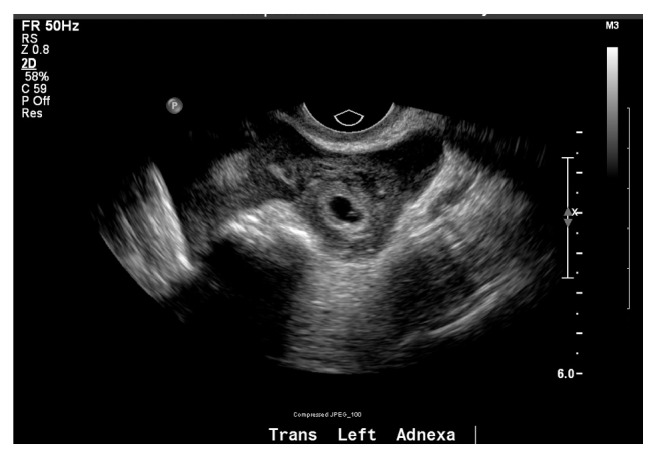
TVUS image of left adnexa with embryonic structures consistent with an ectopic pregnancy.

The patient subsequently presented to the emergency room the following morning with diffuse lower abdominal pain and vaginal bleeding. TVUS identified what appeared to be a corpus luteal cyst in the right ovary (Figure 1) and a likely ectopic pregnancy in the left adnexa (Figure 2) with a small amount of complex free fluid within the cul-de-sac. Her abdominal exam was significant for involuntary guarding of the lower quadrants bilaterally with diffuse tenderness. After discussion with the patient regarding our concern for ruptured ectopic pregnancy, the patient was amenable with the plan of proceeding with a laparoscopic unilateral salpingectomy.

**Figure 3:**
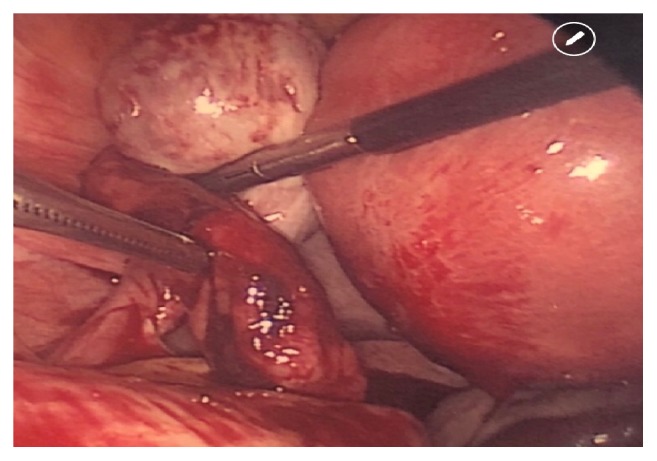
Left tubal ectopic pregnancy (active bleeding, treated with salpingectomy).

**Figure 4:**
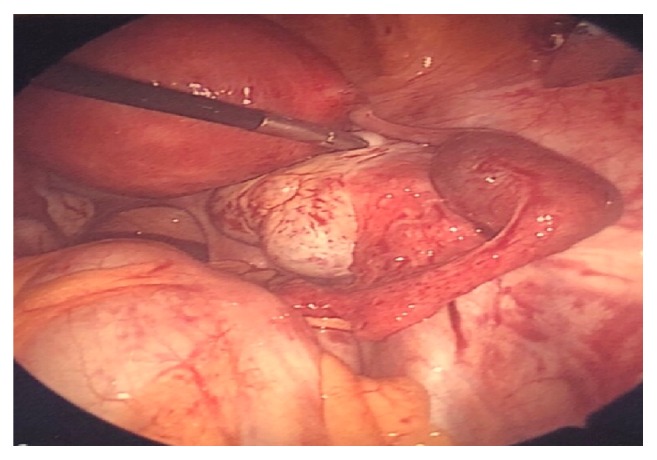
Right tubal ectopic pregnancy (treated with MTX).

A diagnostic laparoscopy was performed which revealed moderate hemoperitoneum upon abdominal entry. On pelvic survey, the left fallopian tube was noted to have a dilated distal portion, approximately 2cm in diameter with active bleeding, consistent with a ruptured left ectopic versus tubal abortion (Figure 3). Notably, the mid-portion of the right fallopian tube appeared dilated at the junction between the isthmus and the ampulla, about 3cm in diameter, without evidence of rupture or bleeding, which was concerning a concurrent second ectopic pregnancy (Figure 4).

The surgeons were then faced with a difficult decision regarding management of the unruptured contralateral tube. The patient's husband (and power of attorney) subsequently became involved with all decisions regarding the patient's plan of care. A left salpingectomy was essential given the abnormal left fallopian tube with active bleeding. Options for management of the contralateral tube were presented: right salpingectomy, right salpingostomy with Methotrexate (MTX) administration, or MTX administration alone without surgical intervention on the right fallopian tube. After thorough risk-benefit consideration, as well as intraoperative consultation with the patient's Reproductive Endocrinologist, the decision was made to retain the right fallopian tube and proceed conservatively with MTX administration (single-dose regimen of 50 mg/m^2^) due to the patient's likely desire to preserve her fallopian tube.

The patient had an uneventful recovery and her day 4 and day 7 *β*-hCG values confirmed an appropriate decline in *β*-hCG levels after MTX injection. The *β*-hCG level had dropped to a nonpregnant level by approximately three weeks following MTX administration. Histology of the left fallopian tube included the presence of chorionic villi, which confirmed the diagnosis of a left ectopic pregnancy. Repeat TVUS one-month following the surgery was normal without evidence of right tubal dilatation. Approximately 14 weeks after surgery, the patient had a repeat TVUS which revealed a single viable intrauterine pregnancy, which was conceived spontaneously.

## 3. Discussion

The general incidence of single ectopic pregnancies varies by study but according to the Center for Disease Control and Prevention accounts for about 2% of pregnancies [5]. Despite improvements in early detection and management modalities, ruptured ectopic pregnancies still account for a significant percentage of pregnancy-related mortality. Evolving data suggests that conception from ART is an independent risk factor for the development of an ectopic gestation. In literature review, studies describe a higher incidence of ectopic pregnancies after various methods of ART, ranging anywhere from 2.2 to 4.5% of ART cycles compared to spontaneous conception [6, 7]. In selected groups of patients with tubal infertility, the incidence of ectopic pregnancies after IVF may be as high as 11% [8]. A recent retrospective cohort study published by the Zhengzhou Reproductive Medical Center analyzed the incidence of ectopic pregnancies in their IVF and IUI cycles over the preceding six years [9]. The overall ectopic rate was 3% in both subgroups, suggesting an increased ectopic risk when comparing ART with spontaneous conception. Bilateral, or heterochronic, ectopics are the rarest form of ectopic pregnancy. We have witnessed a 3-fold increase in diagnosed bilateral ectopic pregnancies over the past several decades, in part due to the increasing use of ART technology [10].

When managing bilateral ectopic pregnancies, important issues arise with regard to detection and treatment. Primarily, trending the *β*-hCG values in bilateral ectopic pregnancies has not been shown to be an effective diagnostic practice [11]. Additionally, in most published case reports on this topic, early ultrasound use typically fails to make a diagnosis of bilateral tubal involvement. In a review of 16 case reports on bilateral ectopic pregnancies after ovulation induction, both ectopic pregnancies were identified by ultrasound imaging in only 6 of the cases prior to surgical intervention [12]. Accordingly, in a review by de los Rios et al. only 2 of 42 bilateral ectopic pregnancies were accurately diagnosed by ultrasound [13]. Commonly, ultrasound imaging identifies one ectopic pregnancy, which precipitates further investigation and subsequent management. Our case highlights this discrepancy between ultrasound results and intraoperative findings. Ultrasound may not be necessary to make the diagnosis, and patients with significant risk factors should be counseled on the possibility of bilateral ectopic pregnancies, and decisions regarding the management algorithm should ideally be decided before surgery ensues. As a majority of bilateral ectopic pregnancies are diagnosed intraoperatively, inspection of both fallopian tubes should be standard of care in any ectopic case where the patient has risk factors for multiple gestations.

Previous studies have suggested that the same options for unilateral ectopic pregnancies be applied for bilateral ectopic pregnancies, including MTX administration or laparoscopy (with either salpingostomy or salpingectomy). However, due to the rare nature of bilateral ectopic pregnancies, there are no published guidelines to help advise management decisions. Also, data is sparse with regard to fertility outcomes and recurrent ectopic pregnancy rate after management of the concurrent ectopic pregnancy. With regard to MTX administration for bilateral ectopic pregnancies, a previously published report described treatment failure using single-dose MTX therapy, as the patient subsequently required surgery [14]. To date, there are no published reports that describe effective MTX dosage or regimen for bilateral ectopic pregnancies.

Bilateral ectopic pregnancies pose a unique dilemma in that both tubes are likely damaged, increasing the risk of future ectopic recurrence. As such, most cases of bilateral ectopic pregnancies are treated with bilateral salpingectomy. For instance, 12 of the 16 cases described by Zhu et al. involved bilateral salpingectomy [12]. In our case, the power of attorney was counseled about potential reproductive options. It was discussed that both fallopian tubes would likely have underlying damage regardless of the chosen treatment option. Discussion continued where bilateral salpingectomies would guarantee the need for IVF while a retained damaged tube would increase the rate of recurrent ectopic pregnancy. The only option that would maintain the possibility of spontaneous intrauterine pregnancy would be to retain the nonruptured tube and to treat medically or with salpingostomy. However, with this approach, if ART was used the Reproductive Endocrinologist would likely recommend IVF in order to decrease the risk of a repeat ectopic pregnancy. This complex and rare situation emphasizes the importance of thorough evaluation of treatment options while utilizing a patient-centric approach.

When deciding between salpingostomy and salpingectomy, it is important to consider the potential impact on future fertility and subsequent ectopic pregnancy risk. RCTs comparing the two techniques in unilateral ectopics have not found significantly different rates for subsequent intrauterine pregnancies or repeat ectopic pregnancies. However, cohort studies have shown higher pregnancy rates for salpingostomy, including both intrauterine and ectopic rates [15]. Surgical management by bilateral salpingectomy should be recommended for usual indication: the patient is exhibiting hemodynamic instability or tubal bleeding. Salpingostomy or MTX administration may be considered for the remaining tube if IVF is not an option; however the patient should be counseled on the recurrence risk and the possible need for future surgery.

In the absence of established guidelines for the management of bilateral ectopic pregnancies, successful outcomes are required in order to help establish protocols for clinical care. Our case presents a unique approach to the management of the contralateral ectopic using medical therapy alone without salpingostomy while already undergoing a surgical procedure, which helped to preserve the remaining tube and allow for spontaneous pregnancy. Complete treatment was confirmed by a downtrend in the patient's *β*-hCG level to a nonpregnant level. Additionally, patients having a unilateral salpingectomy with Methotrexate for the remaining ectopic may consider performing a HSG remote from surgery in order to determine residual tubal patency. One limitation of our study is that tissue was not extracted from the second fallopian tube; thus histologic confirmation of the second ectopic pregnancy is not available.

In conclusion, we present a case of bilateral ectopic pregnancy which was successfully managed with unilateral salpingectomy and medical management with Methotrexate for the contralateral ectopic. As the use of ART technique may become increasingly more common, we may continue to witness a rising incidence of bilateral ectopic pregnancies. As scarce data is published on this topic, it is essential to investigate innovative diagnostic and treatment modalities in order to promote improved clinical care within this context.
